# Neonatal Encephalopathies: A Clinical Perspective

**DOI:** 10.7759/cureus.4948

**Published:** 2019-06-19

**Authors:** Edgar Andrade, Wilson Chavez, Zakir I Shaikh, Alcy R Torres

**Affiliations:** 1 Pediatric Neurology, Institute of Pediatric Neurosciences of Florida, Ocala, USA; 2 Radiology, Boston Medical Center, Boston, USA; 3 Pediatrics, Surat Municipal Institute of Medical Education and Research, Surat, IND; 4 Pediatrics, Boston University School of Medicine, Boston, USA

**Keywords:** neonatal, seizures, encephalopathies, piridoxine, folate, deficiencies

## Abstract

Seizures are the most acute evident manifestation of central nervous system dysfunction in neonates. The incidence is higher in very low weight neonates, about 58/100 live births, as opposed to full-term infants, estimated about 3.5/100 live births. Neonatal seizures represent the clinical manifestation of a non-specific disorder of cortical cerebral dysfunction, which could lead to permanent brain injury. The etiology is multifactorial and requires a judicious assessment of each clinical scenario. The diagnosis and its management are further complicated as most neonatal seizures may have very subtle or no clinical changes and the diagnosis may be just based on EEG findings, so-called subclinical. The treatment is dependent on the etiology, but early and opportune intervention can prevent further brain damage and improve prognosis. Although early identification and treatment are essential, the diagnosis of neonatal seizures can be further complicated by the clinical presentations, possible etiologies, and treatments. Nevertheless, research studies and clinical evidence have shown that early treatment with anti-seizure medications can change the outcome.

## Introduction and background

Neuropathophysiology: disease process

Seizures are the clinical consequence of depolarization and excessive synchronous discharge of the neurons. Depolarization occurs secondarily to intracellular sodium influx and repolarization is caused by potassium efflux. Maintenance of this balance is linked to the active function of the energy-dependent ATP pump, which reverses this process and regulates the passage of sodium to the extracellular space and potassium to the intracellular space. Excessive depolarization may occur due to one of the following reasons [1]:

- The excessive depolarization of the ATP sodium and potassium pump leading to the inability to maintain a membrane potential. This can be caused by hypoxia, ischemia or hypoglycemia.

- An excessive amount of excitatory neurotransmitters like glutamate, resulting in an excessive synaptic release or decreased reuptake of other neurotransmitters producing depolarization. The hypoxic-ischemic injury will lead to increased excitatory neurotransmitters. 

- Relative deficiency of inhibitory neurotransmitters may cause an increased depolarization rate. The gamma-aminobutyric acid (GABA) receptor is the most important inhibitory neurotransmitter but its concentration is lower in the newborn brain as opposed to the adult. Also, the paradoxical excitatory depolarization of GABA receptors activity has been identified in immature neurons and is secondary to the reversal of the chloride gradient in neonatal neurons.

- Absolute deficiency of inhibitory GABA receptors as it is seen in pyridoxine-dependent epilepsy may contribute to seizures. Glutamic acid decarboxylase requires binding to pyridoxine and pyridoxal phosphate. A state of pyridoxine deficiency leads to decreased pyridoxal 5 phosphate and GABA levels.

- Hypocalcemia or hypomagnesemia affect membrane stability leading to inhibition of sodium transit, resulting in depolarization [1].

Neuroanatomical features

The most important processes of relevance to the manifestation of clinical seizures in newborns are organizational events. The relevant steps include lamination of cortical neurons, axonal and dendritic ramifications, and synaptic collections. Lamination is fully developed in full-term babies. However, the other two steps, neurite overgrowth and synaptogenesis, are rudimentary in the newborn. Such a process is required to provide cortical connectivity to propagate a generalized seizure. The relatively advanced cortical development noted in limbic structures and the connectivity to the diencephalon and brain stem may underlie the frequency of some of the clinical manifestations such as orolingual movement, ocular movements, and apnea as clinical manifestations [1-2].

Neurophysiological hallmarks

The relation between excitatory and inhibitory synapses is a critical factor to determine the capacity of a focal discharge to form and spread to other brain regions. The rates of development of inhibitory synaptic activities are different in the neonatal cortex. Excitatory activity is mediated by N-methyl-D-aspartate (NMDA) and alpha-amino-3-hydroxy-5-methyl-4-isoxazolepropionic acid (AMPA). Both neurotransmitters are the predominant demonstration of excitatory receptors in the neonate with a relative paucity of the inhibitory receptor GABA. Furthermore, the immature brain is characterized by the overexpression of excitatory neurotransmitters as compared with the adult cortex, while inhibition has not reached the adult levels. In addition, NMDA receptors have a prolonged duration of the NMDA-mediated excitatory potential, reduced ability of magnesium to block NMDA receptor activity, diminished inhibitory polyamine binding sites and greater sensitivity to glycine enhancement. Likewise, AMPA receptors are deficient in the GluR2 subunit. The immaturity leads to an increase in the permeability of AMPA receptors to Calcium and therefore enhanced excitation. Contrary to what is expected, early in brain development, the inhibitory neurotransmitter GABA is the major postsynaptic GABA receptor, producing excitation rather than inhibition, leading to a lower seizure threshold [1].

Clinical aspects

Neonatal seizures represent a dysfunction of extracellular hyperexcitability presenting in the first days of life secondary to a congenital inborn error of the metabolism. This group of disorders may involve a dysfunctional protein, an enzymatic deficiency or an excess of a byproduct; usually due to a faulty metabolic pathway [2]. In this article, the most common epileptic encephalopathies are reviewed. In addition, a discussion of recently described channelopathies presenting with seizures early in life is also discussed.

## Review

Pyridoxine dependency epilepsy

Definition

This autosomal recessive disorder was first described by Hunt et al. as a case report of intractable convulsions in an infant [3]. Patients are dependent but not deficient of pyridoxine. It is a disorder characterized by an impairment of the lysine degradation pathway [4]. In normal subjects, lysine is converted to pipecolic acid, which in turn is converted to alpha-aminoadipic semi-aldehyde. This latter compound can be converted by alpha-aminoadipic semialdehyde dehydrogenase (AASDH), better known as antiquitin, to alpha aminoadipate or to delta-piperidine-6-carboxyl (P6C). When AASDH is deficient, alpha aminoadipic semialdehyde or delta piperidine -6-carboxyl are increased. P6C impairs the formation of pyridoxal phosphate, which is the active metabolite of pyridoxine, leading to pyridoxine deficiency. It has been demonstrated that some patients with pyridoxine-dependent seizures may respond to pyridoxal phosphate but not to pyridoxine. Overall, patients experience seizure control with pyridoxine monotherapy and seizure recurrence with pyridoxine withdrawal [5].

Biochemistry

Vitamin B6 is ingested by either consumption of vegetables or meat. Vegetables contain pyridoxine, which is metabolized to pyridoxine phosphate through a kinase. Pyridoxine phosphate can be converted to a pyridoxal 5’phosphate (PLP) through the enzyme pyridoxal-N-phosphate oxidase (PNPO). Certainly, PNPO can also metabolize pyridoxamine (nutrient available in meat) to pyridoxal phosphate. The latter compound is then transported into the cell by a membrane-associated phosphatase. Patients are not pyridoxine deficient but require a lifetime supplementation of pyridoxine. The clinical presentation of pyridoxine-dependent children includes neonatal seizures poorly responsive to anti-epileptic treatments, altered mental status, encephalopathy, irritability, and gastrointestinal symptoms. Developmental delays are common. About 154 cases have been reported in medical literature since the first case in 1954. Baxter et al. reported a point prevalence of 1/687,000 [6]. Other authors have reported one case in 20,000 [7]. A possible fetal onset with intrauterine seizures has also been postulated. The similarities with hypoxic-ischemic encephalopathy make the diagnosis more difficult, and up to 30% of patients in some series can be misdiagnosed as hypoxic-ischemic encephalopathy. About 33% of cases present with atypical symptoms, mainly late seizure onset usually between two months and nineteen months of age with initial seizure control using conventional antiepileptic medications (AED) which is rapidly followed by a pharmaco-resistant state [6]. Patients have multiple seizure types such as clonic, tonic, myoclonic, and infantile spasms. Patients developed seizures resistant to AED and usually, seizures are better controlled after administration of pyridoxine (oral or IV). Intravenous pyridoxine administration is recommended at a dose of 100-500 mg IV with continuous VEEG monitoring as a treatment trial for recurrent seizures or for status epilepticus occurring during the neonatal period without a clear explanation [8]. The patients require EEG monitoring and very good critical care evaluation and management. It is important to always have the patient with suspected pyridoxine-dependent to be admitted to the ICU to be able to challenge response to pyridoxine [9]. For diagnosis, it is essential to document both clinical response and resolution of seizures on EEG monitoring after IV pyridoxine has been administered. The diagnosis is strongly suspected when a neonate is in status epilepticus and responds to the administration of IV pyridoxine. Oral administration is indicated for less frequent seizures [10].

Sometimes, the presentation is more discrete and the patient may experience recurrent seizures but not status epilepticus. Then, the diagnosis is suspected after several weeks or months of using AED with poor response. In this cohort of patients with more subtle symptoms, the diagnosis is confirmed when oral pyridoxine is administered and there is a positive clinical response within the next 3-4 days of pyridoxine administration [10]. Pyridoxine dependence diagnosis is solidified by documenting the resolution of clinical symptoms and subclinical seizures with pyridoxine administration. The pyridoxine dependence state is confirmed by genetic testing. It is also important to confirm that there is no evidence of a pyridoxine deficiency state. In general, a deficiency state is very uncommon, and the treatment plan should rule out a pyridoxine deficiency state. The recommended daily allowance (RDA) in the adult is 2 mg/day and in children is 0.5 mg /day. However, because this is a dependency state, patients require pharmacological doses. It is also important to distinguish a pyridoxine-responsive state from pyridoxine dependence. Patients with a pyridoxine-responsive state do not experience seizure recurrence after stopping pyridoxines. In some patients with a diagnosis of pyridoxine-dependent seizures, the pyridoxine-dependent status was never established because pyridoxine was never withdrawn and may not have biochemical or genetic testing to confirm pyridoxine-dependent state. Therefore, it is safer to consider such patients as pyridoxine responsive and not pyridoxine-dependent because the latter was never established as the patient was maintained on daily pyridoxine prophylaxis [11].

Pyridoxine has six natural sources: pyridoxine, pyridoxamine, pyridoxal, and its accompanying phosphorylated forms pyridoxine phosphate, pyridoxal phosphate and pyridoxamine phosphate. It is important to know that pyridoxine phosphate and pyridoxamine phosphate can be converted into PLP through the action of an oxidase. The RDA is 0.5 mg in infants and 2 mg in children and deficiency is very uncommon. The etiology of this deficiency has evolved extensively. Initial investigations by Scriver et al. in 1960 proposed a genetic alteration in the binding of pyridoxal phosphate to glutamic acid decarboxylase and affecting the synthesis of glutamic acid may be the cause [12]. Decreased GABA and increased glutamic acid results were identified and it was postulated as the possible cause of irritability and seizures. Although pharmacological doses of pyridoxine improved the symptoms, this theory was later disproved. Later, Battaglioli et al. postulated a possible genetic alteration of the isoforms of Glutamic Acid Decarboxylase (GAD), either GAD 65 and GAD 67. However, no genetic alteration has been demonstrated [13]. Most recently, elevated pipecolic acid in plasma and CSF were documented by Plecko et al. in patients with pyridoxine dependence state [14]. Pipecolic acid levels persist elevated despite treatment with pyridoxine for years. This detection of pipecolic acid may indicate an indirect marker of pyridoxine dependence. Thus, documenting an elevated pipecolic acid will support the diagnosis of pyridoxine dependence and preclude the need for a diagnostic pyridoxine withdrawal.

Most recently, Struys et al. identified alpha-aminoadipic semialdehyde (AASA) as a more specific biomarker because it is detected in plasma, CSF, and urine [15]. AASA is in equilibrium with its shift base P6C and alpha aminoadipic semi-aldehyde which reversibly conjugates with pyridoxal phosphate leading to an inactive form. Hence, these patients have a deficient intracellular pyridoxal phosphate status. P6C binds all co-factors within the cell leading to various alterations of different intracellular metabolic pathways and causing a pyridoxine-dependent state.

In general, L-pipecolic acid and its metabolites P6C and alpha amino-adipic semi-aldehyde are byproducts of L-lysine metabolism [16]. Further, Mills et al in 2006 proposed that although the patients are pyridoxine deficient, high AASA levels are secondary to mutations in alpha-amino-adipic semi-aldehyde dehydrogenase (ALDH7A1 ) [17]. Positive ALDH7A1 mutation status has been consistently identified in several neonates with pyridoxine-dependent seizures.

ALDH7A1 mutations have been characterized in a number of cases, including both early and late onset presentations [17]. However, a firm phenotype-genotype correlation has not been established. Moreover, there have been cases having normal biomarker levels and no mutation, suggesting other causes of pyridoxine dependency. In general, it is believed that late-onset cases have a better prognosis. Early diagnosis and treatment may improve developmental outcome [18]. Some other patients have several complications including profound mental deficiency despite early treatment. In terms of treatment, a dose of 5 mg/kg/day may control seizures but up to 18 mg/kg/day may improve development. The total dose is usually not more than 500 mg per day in order to prevent pyridoxine-related toxicities such as peripheral neuropathy when receiving lifetime pyridoxine. For patients with pyridoxine-dependent epilepsy, is also recommended to use folic acid 3-5 mg/Kg/day as both diseases may present similarly [19].

Glucose transporter type I deficiency 

This disorder was first described by DeVivo et al. and is characterized by defective glucose transport across the blood-brain barrier. This abnormality leads to persistent low cerebrospinal glucose which causes seizures and developmental delay. The diagnosis is suspected by the identification of significant hypoglycorrhachia in the setting of normal blood glucose. Genetic testing is available to confirm the diagnosis. The treatment is based on the use of a ketogenic diet [20].

Biotinidase deficiency

Definition

The patients are born with an inability to cleave biocytin, leading to a deficiency of multiple carboxylases. There is extensive genetic heterogeneity and variants may include partial biotinidase deficiency with variable penetrance [21].

Biochemistry

There are two forms of biotin; dietary free biotin and biotin that is ingested in any regular diet as protein bounded. First, biotin is converted to biocytin. However, biocytin must become free biotin through a reaction that involves biotinidase. Free biotin is a cofactor for the conversion of apo-carboxylase synthetase that converts apocarboxyl to hydro-carboxyl. Hence, the lack of biotin cause an elevation of apocarboxyl [21].

Clinical Presentation and Treatment

Symptoms present early in life with episodic metabolic acidosis, alopecia, peri-oral rash, eczema, seizures, ataxia, hypotonia, and developmental delay. Untreated patients will experience hearing loss, optic atrophy and subsequent visual loss which is permanent if not treated early. Very frequently, patients develop lactic acidemia and propionic acidemia. This cohort experience a good response to biotin (10 mg/day) and patients will require a lifetime supplementation. However, sensory-neural hearing loss may persist [22].

Most states test for biotinidase deficiency during the neonatal newborn screening; several patients that immigrate to the US may not have been tested for biotinidase deficiency. Biotinidase deficiency may present later in life with progressive spastic paraparesis and rash. The first clinical presentation in the majority of patients is seizures in the first six months of life, including infantile spasms, myoclonic seizures or generalized seizures. Thus, biotinidase deficiency must be ruled out in patients with unexplained seizures [23].

Developmental delay, epilepsy, neonatal diabetes: DEND

Definition

This potassium channelopathy is characterized by developmental delay, epilepsy, and neonatal diabetes. The symptoms present in the first days of life. The pregnancy and delivery are usually unremarkable and there is no family history of neurological disorders. The patients present with intractable hyperglycemia, very low insulin secretion and undetectable islet cell antibodies causing neonatal diabetes. The patients experience hyperglycemias that respond poorly to insulin. Neonatal seizures are seen in the first days of life and may also evolve to infantile spasms. EEG shows typical hypsarrhythmia and bursts suppression pattern. Developmental delays are profound and include hypotonia, poor head or trunk control, unable to sit or walk positive cortical thumbs, hyperreflexia, athetosis and overextended legs. Patient’s response to antiepileptic medications is poor. Hyperglycemia improves with oral hypoglycemic agents such as glibenclamide [24]. Along with the resolution of hyperglycemia, developmental delays are reversed. Patients can eventually roll prone to supine, lift head up, sit with minimal support, open hands, grasp spontaneously and can fixate and follow if the diagnosis and treatment are established promptly. The diagnosis is established by sequencing of genes KCNJ11 and ABCC8 that encodes the subunits of the potassium ATP channel Kir6.2SUR 1. This missense mutation is located within the trans-membrane channels [25]. The expression of the mutant potassium ATP channel in Xenopus oocytes has revealed an increased rate of spontaneous opening and reduced ability of intracellular ATP to close channels. In normal subjects, sodium and potassium channels have opposite effects on the membrane potentials. The channels are in the membrane cell. When sodium channels open, allowing ions to cross across the membrane along the concentration gradient. When the sodium-potassium pump opens, it drives potassium into the cell and sodium out of the cell. When sodium channels open, sodium comes into the cell and depolarizes the membrane. If potassium channels open, then potassium comes out of the cell and cause repolarization.

The potassium ATP channel is an energy gatekeeper in the pancreatic beta cells and the neurons. In a hypoglycemic state, the potassium channel opens, and calcium and sodium channels closed. Therefore, cell membranes are hyperpolarized, and potassium leaves the cell, leading to no insulin release. In a hyperglycemic environment, glucose comes into the cell, causing increased ATP and decrease magnesium ADP (MG ADP). These changes close the potassium ATP channels, and the membrane is depolarized, causing opening of calcium channel, thus, causing calcium to enter into the cell and produce an active release of insulin.

In patients with mutant potassium ATP channels, the channel uncouples insulin release from elevated serum glucose and intracellular ATP. In the situation of hyperglycemia, glucose enters the cell, causing increased ATP, decreased MG-ADP and open K-ATP channels. Then, calcium channels close and there is no insulin release. Sulfonylureas drugs block potassium channels, promoting insulin release [26]. It is not well understood how mutations of potassium ATP channels cause developmental delay and epilepsy. It has been postulated a loss of synaptic inhibition. The channel is highly expressed by hippocampal interneurons and pyramidal cells. K-ATP openers may potentially hyperpolarize hippocampal interneurons but have little effect on pyramidal cells. Individuals with excessively active mutant K-ATP may have compromised interneurons, causing seizures. It is believed that potassium channels ATP is a protective gatekeeper of hippocampal interneurons. Thus, in patients with normal metabolism, when glucose is metabolized within the cell, ATP is increased, and Mg-ADP is decreased, causing the K-ATP channels to close and membrane depolarization to a point of permissible action potentials and neurotransmitter release and synaptic GABA release. When mutant K-ATP channels are overactive, like what is seen during metabolic distress, the channels are open, potassium leaves the cell, the membrane is hyperpolarized, and the action potential firing affects neurotransmitters release, causing impairment of GABA release.

It has been identified in some patients K-ATP mutations that reduce sulfonylurea blocker efficacy and preventing treatment by currently available agents. Also, the sulfonylureas levels in the brain are reduced by active export. It is unknown if early identification and treatment with sulfonylureas reduced the degree of neurological impairment. Sulfonylurea responsiveness can be studied functionally by patch clamp electrophysiology in subjects with a positive mutation status [27].

Creatine synthesis defects

Definition

This group of disorders was first described in 1994 and is characterized by a deficiency of either guanidinoacetate N-methyl transferase (GAMT) or arginine-glycine aminotransferase (AGAT) or due to a transporter defect. The guanidinoacetate levels are elevated if GAMT is deficient, low if AGAT is deficient, and normal if it is a transporter defect.

Biochemistry

Creatine is formed via arginine through AGAT that catalyzes the conversion of arginine to guanidino-acetate (GAA). For this reaction to occur, glycine is a cofactor and is converted to ornithine. Then, GAMT catalyzes the conversion of GAA to creatine. Creatine must be transported into the brain through a creatine transporter 1. The receptor can be defective and creatine is not actively transported through the blood-brain barrier [28].

Clinical Presentation

Patients present with developmental delay, predominantly language delay, febrile seizures, and febrile status. Eventually, they developed myoclonus, head drops, and generalized tonic-clonic activity. The neurological exam reveals significant language delay such as single-word or two-word utterances, truncal hypotonia, increased appendicular tone, and dystonic postures. Seizure types include generalized, atypical absences, myoclonic seizures and drop attacks. There are subtle differences among the three types of creatine synthesis defects. The patients with GAMT deficiency present with developmental delay early in life and severe psychomotor retardation, developmental regression, autism, hypotonia, epilepsy, and movement disorder. The brain magnetic resonance imaging (MRI) of patients with creatine synthesis disorder reveals signal changes in the globus pallidus. The patients with AGAT deficiency experience reduced somatic growth, early developmental delay, intellectual deficiency, and epilepsy. The patients with creatine transporter defects present with intellectual deficiency, hypotonia, and epilepsy [29].

Brain magnetic resonance spectroscopy (MRS) in subjects affected with creatine synthesis deficits shows increased creatine to choline ratio, reflecting a low serum choline level and a normal creatine level. Also, the creatinine /creatine ratio in serum is elevated. EEG shows disorganization and poorly formed background as well as generalized and multifocal spikes. Treatment involves the use of creatine supplementation for the group of patients with GAMT and AGAT deficiencies. For the group of patients with only GAMT deficiency, the treatment is based on arginine restriction and ornithine supplementation [30].

Hyperekplexia

This disorder is rather an epilepsy mimic and the clinical presentation is fetal seizure-like events and /or neonatal seizure-like events. This may include fisting, increased muscle tone, rhythmic movements of extremities, increased muscle tone, choking and apneic spells. The patients usually demonstrate an excessive startle response. They are sensitive to a tap of the nose. The disorder is secondary to a defect in the glycine transporter or a mutation in the glycine inhibitory receptor (GLRA 1). The attacks may be prevented by sudden flexion of head and limbs. Sudden death may occur because of choking episodes or obstructive apnea [31]. The treatment is usually based on the use of benzodiazepines such as clonazepam, ativan, or diazepam. Patients may experience a gradual improvement with age. Children that receive treatment demonstrate improvement of the developmental milestones are more interactive, muscle tone is better, and can perform several developmental skills for their age [32].

Disorders of serine biosynthesis

Definition

This group of disorders is characterized by low plasma and CSF serine levels. Patients present with congenital microcephaly and psychomotor retardation. Seizures usually manifest after 4 months of age with infantile spasms and may develop generalized epilepsy later in life if diagnosis and treatment are delayed.

Biochemistry 

Glucose is converted to 3-phosphoglycerate (3PDG), which catalyzes phosphoglycerate to 3-phosphohydroxypiruvate and to 3 phosphoserines. Then, 3-phosphoserine is converted to L-serine. L-serine is a cofactor of multiple reactions, predominantly in the conversion of methionine to homocysteine and the conversion of tetra-hydro folate to 5, 10-meta-tetrahydrofolate [33]. The diagnosis of serine deficiency usually is identified with plasma amino acids evaluation but sometimes is not possible and serine levels should be determined in plasma and CSF.

Clinical Presentation

The patients resemble any other cases of a congenital static encephalopathy, such as congenital toxoplasma, cytomegalovirus, herpes, or rubella infection. However, serine biosynthesis disorder must be kept in mind when considering a newborn with unexplained congenital microcephaly and psychomotor retardation. There are two possible enzyme deficiencies that can cause serine deficiency: 3-phosphoglycerate dehydrogenase (3-PGD) and 3-phosphoserine phosphatase (3PSP) [34]. The treatment involves lifetime serine supplementation at a dose of 400-600 mg/kg/day and glycine at a dose of 200-300 mg/kg/day. The outcome is usually normal if the diagnosis is suspected and established promptly and treatment is started after birth and maintain for life without interruptions.

Folinic acid-responsive seizures

Definition

First described by K Hayland in 1995 as seven cases with neonatal seizures unresponsive to AED and poor response to pyridoxine but responsive to folinic acid.

Biochemistry

Research studies have shown unidentified peaks in liquid chromatography analysis. Other authors have also identified the same ALDH7A1 mutation. Thus, both conditions Pyridoxine-dependent and folinic acid responsive seizures are allelic of the same mutation. We do not know how folinic acid may affect the response to treatment of pyridoxine-dependent seizures. It is unclear if a low lysine diet would improve the outcome of these patients. Antiquitin dysfunction and ALDH7A1 mutations have been demonstrated in such cases [35].

Treatment

The recommended dose of folinic acid is 3-5 mg/kg/day by mouth. Folinic acid can cause gastrointestinal upset and the dose must be modified to improve compliance [36].

Cerebral folate deficiency

Definition

This disorder is characterized by low tetra-hydro-folate levels in CSF.

Biochemistry

CSF will reveal low 5-meta-tetrahydrofolate levels with normal mono-amino metabolite levels. Seizures altered mental status and involuntary movements improve if the diagnosis and treatment are established promptly. Some patients may develop an antibody against the folic receptor [37].

Clinical Presentation and Treatment

The patients present in the first months of life with irritability, arrested head growth development, seizures, ataxia, pyramidal tract signs, psychomotor retardation and a constellation of movement disorder that includes ballismus and chorea-athetosis. Generalized tonic-clonic activity usually is first seen after the first year of life. The EEG reveal disorganized background activity, polymorphic spikes and slow waves discharges. During early childhood, patients developed optic atrophy and cortical blindness. Patients with cerebral folate deficiency also experience progressive loss of motor milestones, followed by motor and visual impairment. Patients develop seizures in the first months of life, accompanied by impairment in motor skills. Later in life, difficulties with gait and balance are evident. The diagnosis is made by checking tetrahydrofolate levels in CSF. Although vitamin B12 and folic acid levels are usually normal, patients only experience a deficit in transporting folate in the brain due to a faulty transporter. Antibodies against the receptor are now commercially available. Therefore, patients usually respond to folinic acid in divided doses because the half-life of folinic acid is about 8 hours. The absorption is impaired so serial monitoring with CSF studies is recommended. We usually repeat a spinal tap every 3 months but taper once the patient is doing well and the parameters stable. We have followed one patient with secondary deficiency due to a mutation that prevents the transport of folic acid from the gut to the blood, who is doing very well in school, receives two daily intra-muscular doses of folinic acid for life. However, improvement in cognitive skills and major developmental delays may not be possible in all children [38].

Pyridoxal 5-phosphate (PLP)-responsive neonatal epileptic encephalopathy

Definition

This group of patients presents with neonatal encephalopathy and seizures that respond to pyridoxal phosphate but does not respond to pyridoxine.

Biochemistry

Studies have revealed an elevation of 3 methoxy tyrosine in CSF, decreased homovanillic acid and vanillactic acid in urine, decreased CSF levels of 5-hydroxyindolacetic acid and increased levels of threonine and glycine. Such findings indicate a deficiency of threonine dehydrogenase and a glycine cleavage enzyme suggesting a pyridoxal phosphate dehydrogenase deficiency [39].

Clinical Findings and Treatment

The diagnosis is based in testing CSF for neuro-transmitters metabolites and amino acids, serum amino-acids, pipecolic acid, and AASA, urine for vanillactic acid and alpha AASA, and genetic testing to evaluate ALDHN 7A1 or PNPO genes. 

The recommended pyridoxal phosphate dose is 30-50 mg/Kg/day divided into 4-6 doses to attain a good outcome. Atypical presentations are a common and high index of suspicion should be maintained. Neurodevelopmental prognosis is variable but early diagnosis and treatment are critical [40].

Hyperinsulinism-hyperammonemia

This form of congenital hyperinsulinism has been identified to be secondary to a mutation of the enzyme glutamine dehydrogenase. Symptoms include recurrent hypoglycemia leading to seizures during episodes of fasting or high protein meal intake, epilepsy, learning disability and significant behavioral symptoms, including short attention span and hyperactive [41]. The disorder is secondary to a mutation of glutamate dehydrogenase enzyme. The treatment involves the combination of AED and carbonic anhydrase inhibitors such as diazoxide. This medication promotes hyperglycemia which stimulates potassium channels and inhibits insulin release [42].

Biopterin synthesis disorders

Definition

Biopterin synthesis disorder is a disorder of recycling or synthesis of Tetrahydrobiopterin (BH4) and presents in early infancy with uncontrollable seizures [43]. This disorder is a subtype of phenylketonuria, a disorder characterized by elevated phenylalanine (PHE). 

Biochemistry

The overall metabolic pathway involves the conversion of Guanidine-Triphosphate (GTP) to the final product Tetrahydrobiopterin (BH4) through the action of GTP hydrolase 1. First, GTP is converted to dihydro-neopterin triphosphate through a GTP cyclohydroxylase (GTPCH). Second, dihydro-neopterin is converted to 6-pyruvoyl-tetrahydropterin (6-PTH) through a 6 pyruvoyl-tetrahydropterin synthase (PTPS). Then, 6-PTH is converted to BH4 through the action of sepiapterin reductase.

BH4 is cycled to dihydro-biopterin through a dihydropterin reductase (DHPR). Of note, BH4 participates in the conversion of tryptophan to 5 hydroxyl-tryptophan through tryptophan hydroxylase. Also, BH4 participates as a cofactor in the conversion of tyrosine to L-dopa through tyrosine hydroxylase. Finally, BH4 participates in the conversion of PHE to tyrosine through phenylalanine hydroxylase. Bh4 is an essential cofactor to produce monoamines, including catecholamines and serotonin.

This group of BH4 disorders can be classified as of whether PHE levels are elevated or normal. Subjects with elevated PHE include pyruvoil-4Hp-pterin synthase (PTPS) deficiency, autosomal recessive GTP cyclohydroxylase (GTPCH) deficiency, dihydropteridine reductase (DHPR) deficiency, and pterin-carbinolamine reductase (PCD) deficiency. On the contrary, autosomal dominant GTP cyclo-hydroxylase deficiency or sepiapterin (SR) deficiency will not affect PHE levels.

Impaired synthesis of dopamine, serotonin, epinephrine, and norepinephrine with normal PHE levels is seen in subjects with deficiency of GTP cyclohydroxylase, dihydropteridine reductase, pterin -carbinolamine reductase, pyruvoyl-4H-pterin synthase, GTP cyclohydrolase, sepiapterin, and BH4 reductases. Folate antagonists are well known to exacerbate symptoms in subjects with BH4 deficiency and affected patients easily decompensate [44].

Clinical Findings and Treatment

Most patients with BH4 deficiency present with myoclonic seizures, microcephaly, dystonia, intellectual impairment in the moderate to severe range, muscular rigidity and drooling. Brain MRI reveals cerebral atrophy and an increased signal of the white matter. In addition, patients with DHPR deficiency usually develop basal ganglia calcifications. Conversion of sepiapterin to BH4 through a dihydrofolate reductase is a salvage pathway for patients affected with deficient BH4 synthesis. Therefore, some degree of improvement in basal ganglia calcifications can be seen using folinic acid supplementation [45].

Although is beyond the scope of this review, deficiency of GTP1 hydroxylase is the cause of Segawa disease, dopa-responsive dystonia characterized by decreased synthesis of the catecholamines, dopamine, and norepinephrine. 

The treatment involves using tetrahydrobiopterin supplementation, neurotransmitters precursors, mono-amino-oxidase inhibitors and catechol-oxy-methyl transferase inhibitors.

Pyruvoyl-tetra-hydropterin synthase deficiency (PTHD)

This is the most common type of biopterin synthesis disorders. The subjects have a deficit in the synthesis of biopterin. Thus, urine pterin is low and is the most important distinguishing finding of the disorders of the synthesis of biopterin. In patients with other types of tetrahydrobiopterin disorders, urine pterin is high. Usually, these patients are given a diagnosis of phenylketonuria in the neonatal period because PHE levels are high. However, urine pterins are normal in patients with PKU. Subjects affected with PTHD deficiency have low urine pterins. Clinically, subjects present with a chronic rash predominantly over the thigh areas, knee pains, gait abnormalities, fluctuating degrees of weakness and stiffness and cognitive impairment but a relatively normal mental status. Symptoms may exacerbate with the use of folic acid inhibitors. The diagnosis is confirmed by measuring blood levels of the enzyme pyruvoyl-tetrahydropterin synthase.

Potassium channel encephalopathies

KCNQ2 Encephalopathy

This is the most common neonatal potassium channel encephalopathy, representing about 10% of this cohort. Patients experience new onset convulsions in the first months of life. There are two forms: early onset and late onset. Early-onset patients usually have seizures in the first three to five days of life. Late-onset patients usually have seizures by three months of age. Mutations in the KCNQ2 and KCNQ3 potassium channels have been identified [46]. The EEG reveals a burst suppression pattern which may evolve into a multifocal interictal pattern. MRI shows hyperintensities in the basal ganglia and thalamus.

Malignant Migrating Partial Epilepsy of Infancy

Patients with this potassium channelopathy have demonstrated the presence of the KCNQ1 mutations. However, additional studies have also shown a mutation of the SCN1 channel, which is a sodium channel that is affected in patients with severe myoclonic epilepsy of infancy (Dravet disease). The seizure onset is usually before six months of age and seizures migrate between either hemispheres. The development is usually poor, and patients typically have a moderate to profound range of mental retardation. This mutation is also seen in subjects with autosomal dominant nocturnal frontal lobe epilepsy [47].

Early Epileptic Encephalopathy with Burst Suppression Pattern

Formerly known as Otahara syndrome, patients with this disorder present, this disorder present in the first 2 to 3 months of life with tonic spasms resembling West syndrome. Babies are profoundly delayed, and the EEG reveals 1-2 hertz generalized spike and polyspike with several seconds of diffuse attenuation of the background and consistent with a burst suppression pattern. Brain MRI may reveal hemi-megalencephaly, frontal atrophy, and thin corpus callosum or other structural changes. Several mutations have been identified, including syntaxin binding protein 1. In addition to infantile spasms, patients may also have a non-epileptic movement disorder. A genetic mutation may be identified in up to 10-20 % in neonates with early infantile Epileptic encephalopathy.

STXBP1 Encephalopathy

This neonatal encephalopathy is characterized by early onset seizures, choreiform movement disorder, and dyskinesia. Older children demonstrate frank hemiballismus. The usual onset is about 18 months of age. The EEG reveals findings ranging from a burst suppression pattern to lateralized epileptiform activity [48].

CHD2 Encephalopathy

This neonatal epileptic encephalopathy presents in the first days of life with seizures. It has been identified as a chromosomal helicase DNA binding protein 2 due to a 15q26.1 deletion. Patients develop a myoclonic and photosensitive epilepsy, multiple seizure types and profound mental retardation, fitting the electro-clinical diagnostic criteria of Lennox-Gastaut syndrome. Interictal EEG reveals a generalized slow and spike and wave pattern [49].

SYNGAP 1 Encephalopathy

This disorder represents 1% of the group of patients with Potassium channel encephalopathies and the onset is around 14 months of age. Patients present with recurrent seizures and profound mental retardation. The EEG reveals a slow spike and waves epileptiform activity.

Other Mutations

A de novo heterozygous missense mutation (c.3979A>G; p.Ile1327Val) in SCN8A (voltage-gated sodium-channel type VIII alpha subunit) gene has been described as the cause of neonatal seizures, multiple anomalies and movement disorders [50].

Other potassium channel encephalopathies

Several mutations in the potassium channels have been identified as the cause of epileptic encephalopathy presenting in the first month of life. Examples of such potassium channels mutations include STXBP1, CDKL5, KCNQ2, and CHD2.

## Conclusions

Although advancements have been made in the level of monitoring in recent years, these are not available to everyone. However, research studies and clinical evidence have shown that early treatment with anticonvulsant medications can change the outcome and improve prognosis. The availability of new, especially more effective drugs has been slow. Advances in genetic and metabolic diagnosis, especially of curable or preventable disorders, have been the greatest progress in recent years.
